# Low Levels of Aflatoxin B1, Ricin, and Milk Enhance Recombinant Protein Production in Mammalian Cells

**DOI:** 10.1371/journal.pone.0071682

**Published:** 2013-08-05

**Authors:** Reuven Rasooly, Bradley Hernlem, Mendel Friedman

**Affiliations:** 1 Foodborne Contaminants, Agricultural Research Service, United State Department of Agriculture, Albany, California, United State of America; 2 Produce Safety and Microbiology Research Units, Agricultural Research Service, United State Department of Agriculture, Albany, California, United State of America; Centro de Investigación en Medicina Aplicada (CIMA), Spain

## Abstract

Gene expression in transduced mammalian cells correlates with virus titer, but high doses of vector for gene therapy leads to toxicity in humans and in animals. Changing the optimal tissue culture medium by adding low levels of environmental stressors, such as 1 µM of the fungal toxin aflatoxin B1 (AFB1), 1 ng of the castor bean protein toxin ricin, or 1% reconstituted milk, enhances transcription and increases production of proteins in transduced mammalian cells as demonstrated by production of the following three recombinant proteins: firefly luciferase, β-galactosidase, and green fluorescent protein (GFP). Higher concentrations of the stress-producing substances damage the cells beyond recovery, resulting in inhibited gene expression and cell death. We also evaluated the effect of the stressor substances on the enhanced infectivity of virus. The presented findings extend methods for large-scale transient recombinant protein production in mammalian cells and suggest that it may be possible to reduce the cytotoxicity of the adenovirus by reducing the virus titer without adversely affecting gene expression levels.

## Introduction

Therapeutic recombinant protein expression is widely used in medicine and agriculture [1]. The annual global market in 2011 for recombinant proteins was estimated at more than 102.4 billion US dollars [2]. Currently about 60% of all recombinant pharmaceutical proteins are produced in mammalian cells, mainly because mammalian cells can post-translationally modify proteins, and thereby significantly enhancing protein bioactivity. It seems that the expression levels in mammalian cells are generally lower than in bacterial systems. Thus, there is a need to increase the yield of recombinant proteins expressed in mammalian cells, and one objective of the present study is to meet this need.

In this study we used adenovirus as a vector, widely used in gene therapy, to introduce recombinant DNA to mammalian cells. Previous investigators reported a dose- dependent toxicity in humans and in animals receiving high doses of this vector [3], [4], [5]. A further objective of this study was to minimize the cytotoxic effects of the virus by reducing the virus titer without reducing the gene expression levels.

In a previous study, we have demonstrated that adding low concentrations of the olive compound 4-hydroxytyrosol or the commercial olive powder isolated from olive juice called Hidrox-12 enhanced the growth of the bacterial pathogen *Staphylococcus aureus* [6]. By contrast, higher concentrations of these natural products were bactericidal against the pathogen. We theorized that the low levels of the antimicrobial formulations slightly disrupt the normal cell environment and injure the bacterial cell.

We also hypothesized that the bacteria react to these stress stimuli to adapt to the environmental changes and disruption by enhancing transcription of genes that alter cell physiology to increase levels of protective molecules and enhance the capability of cellular systems involved in adaptation, cell survival, and bacterial growth. When the concentrations of the antimicrobials are increased, the bacteria cannot overcome the damaging effect of the antimicrobials, resulting in cell death.

These observations imply that it may be possible to change the optimal cell culture medium and expose mammalian cells to moderate environmental stress to induce the biosynthesis of bioactive recombinant proteins.

In this present study, we demonstrated that changing the optimal tissue culture medium with added low concentrations of milk or the known toxins aflatoxin B1 (AFB1) and ricin results in enhanced recombinant protein production, as demonstrated by production of three recombinant proteins tested in transduced mammalian cells. As in the case with the bacteria, increased concentrations of stress substance damage the cells beyond their limits of recovery, resulting in inhibited transcription and cell death.

## Materials and Methods

### Materials

Purified ricin was obtained from Vector Laboratories (Burlingame, CA, USA). Aflatoxin B1 (AFB1) was purchased from Sigma-Aldrich (St. Louis, MO, USA). Nonfat dry milk from Nestlé Carnation (Vevey, Switzerland) was dissolved in distilled water to form 0.1–5.0% solutions. Human Embryonic Kidney 293 cells (HEK293) (ATCC CRL-1573) and Vero African Green Monkey adult kidney cells (ATCC CCL-81) were obtained from American Type Culture Collection (Manassas, VA, USA).

Primers for Adeno-X were obtained from Clontech (Mountain View, CA) and human GAPDH primers from R&D (Minneapolis, MN).

### Effect of heat on AFB1 activity

To determine the effect of heat on AFB1 activity, samples of Dulbecco's Modified Eagle Medium (DMEM) (500 µl) spiked with increasing concentrations of AFB1 in tightly screw-capped Eppendorf tubes (1.5 ml) were inserted in an Eppendorf Dry Heating Block (Government Scientific Source Inc., Reston, VA, USA) set at 63°C or 100°C for 5 min. The cooled samples were then incubated with Vero cells.

### Cell culture

Vero cell and Human Embryonic Kidney 293 cells (HEK293) were maintained in DMEM medium containing 10% fetal bovine serum (FBS) and 100 units/ml of both penicillin and streptomycin. Cells were trypsinized when ready to harvest.

### Generation of Adenoviral Vectors that Express GFP Gene, Lac-Z Gene or Luc gene

In order to quantify the effect of added stress factors to the optimal cell-culture medium on recombinant gene expression, three different reporter gene vector constructs were prepared. The GFP gene was isolated from the Green Lantern vector (Life Technologies, Grand Island, NY, USA). The bacterial Lac-Z DNA sequence was isolated from a pVS-β-galactosidase plasmid vector (Promega) and the Firefly Luciferase sequence was isolated from pGL3-Basic Vector (Promega). The fragment was purified from gel using a Qiagen Kit and was subcloned into the adenoviral shuttle plasmid between the Cytomegalovirus immediate-early promoter (CMV) or Rous Sarcoma Virus promoter (RSV) and the polyadenylation signal from bovine growth hormone. The plasmid pJM17 containing the full length of the adenovirus genome including a 4.4 Kb sequence of antibiotics resistance gene was co-transfected in HEK293 cells along with the shuttle plasmid containing the reporter gene flanked by the adenovirus E1 sequences. After 10 days, the cytopathic effect appeared, and the transfected cells became round and detached from the plate. The cells were then analyzed for the expression of the reporter gene. An individual plaque of the adenovirus vectors that encode and express the reporter gene was amplified.

### Plaque assays for purification and titration of the adenovirus

Plaque assays depend on the ability of the adenovirus to propagate in HEK293 cells. Six 35 mm tissue culture plates were seeded with HEK293 cells. The cells were incubated at 37°C in a 5% CO_2_ incubator until they were 90% confluent. Serial dilutions were made in DMEM medium supplemented with 2% FBS. The diluted virus was added to the cells. After 2 hr, the medium was removed and replaced with 1× Modified Eagle Medium and 1% sea-plaque agarose (FMC). The agar overlay was added to keep the virus localized after the cells had lysed. After 5 days, plaques were visible, and counted for titer determination after 7 days.

### Quantifying recombinant adenovirus expressing vectors that encode firefly luciferase (Luc), β-glucoronidase (Lac-Z) or green fluorescent protein (GFP) genes

Vero cells were plated on black 96-well plates (Greiner 655090 obtained from Sigma) at 1×10^4^ cells in 100 µl of medium per well. Cells were incubated overnight to allow time for cells to attach to the plate. The cells were then transduced with Ad-GFP, Ad-Lac-Z, or Ad-Luc at a Multiplicity of Infection (MOI) of 100. After 1 hr, samples (either 15 µl of sample in 85 µl of media, or 100 µl of media spiked with toxins) were added to each well and incubated for 24–48 hr at 37°C in a 5% CO_2_ incubator. The sample was removed and cells were washed three times with pH 7.4 phosphate buffered saline (PBS).

Quantification of fluorescence emission by the cells expressing GFP was measured using a 528/20 nm emission filter and 485/20 nm excitation filter in a Synergy HT Multi-Detection Microplate Reader (BioTek, Winooki, VT, USA). The luciferase enzyme activity and quantitative β-glucoronidase expression using Beta-Glo substrate were determined according the manufacturer's instructions (Promega, Madison, WI, USA).

### mRNA analysis

Total RNA was prepared using the Trizol reagent as described by the manufacturer (Life Technologies). RNA was prepared from Vero cells after stimulation with AFB1, ricin or 1% milk. After RNA isolation, first-strand cDNA was synthesized from 1.0 µg of total RNA using reverse transcriptase (Superscript II; Life Technologies) in a 25-µl reaction with oligo-dT (Life Technologies) as a primer. RNA was then degraded by treatment with RNaseH (Life Technologies). Quantitative PCR analysis was performed using 1 µl of cDNA with 0.25 µmol primers in a 20-µl reaction using an Eppendorf Mastercycler ep realplex thermal cycler (Eppendorf, Hauppauge, NY, USA) and Brilliant SYBR Green QPCR master mix reagents (Agilent Technologies, Santa Clara, CA, USA). The amplification program began with a 3 m denaturation step at 95°C followed by 40 cycles with a 94°C denaturation for 30 s, annealing for 30 s at 56°C, and extension at 72°C for 1 m. Upstream and downstream primers for genes of interest were as follows: *GAPDH*, 5′-TGG ACC TGA CCT GCC GTCT-3′, 5′-GGA AGA GTG GGT GTC GCT GT-3′; β-Galactosidase (*LacZ*) 5′- GTT GCA GTG CAC GGC AGA TAC ACT TGC TGA-3′, 5′-GCC ACT GGT GTG GGC CAT AAT TCA ATT CGC-3′; *Luciferase*, 5′-AGA CGC CAA AAA CAT AAA GAA AGG CCC GGC-3′, 5′-TAT AAA TGT CGT TCG CGG GCG CAA CTG CAA-3′.

To confirm specificity of the reaction product during each run, the melting profile of each sample was analyzed using the Eppendorf realplex. The melting profile was determined by holding the reaction at 55°C for 15 s and then heating slowly to 95°C with a linear rate of 0.2°C/s, while the fluorescence emitted was measured. Melting curve analysis was used to ensure that each of the primer pairs described amplified a single product.

The fold change of each gene was calculated by normalization of the gene of interest with the housekeeping gene, GAPDH, using the following formula:
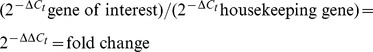



To confirm that each primer pair correctly amplified the sequence of interest, initial PCR products from cDNA were run on an agarose gel (1.5% SeaKem 3∶1), stained with GelRed (Phenix Research, Candler, NC, USA), and viewed by UV transillumination. The method used confirms that a single product of the predicted size was produced.

### Flow cytometric analysis

Vero cells were strained through a 30 µm screen and analyzed on a FACSVantage SE (BD Bioscience, San Jose, CA, USA) flow cytometer equipped with a 100 mW, 491 nm Calypso (Cobolt, Sweden) laser. CellQuest software (BD Bioscience) and FlowJo software (TreeStar, Ashland, OR, USA) were employed for data collection and data analysis, respectively. Vero cells were discriminated from debris by forward and side scatter of laser light. GFP fluorescence was quantified in the Vero cell population using a 530/30 nm bandpass filter.

### Statistical analysis

Statistical analysis was performed with SigmaStat 3.5 for Windows (Systat Software, San Jose, CA). Multiple comparisons among transduced treated cells were made. One-way analysis of variance (ANOVA) was used to compare transduced treated cells (containing increasing concentrations of toxin or milk) to control and transduced untreated cells. The experiments were repeated at least three times, and results with P<0.05 were considered statistically significant.

## Results

### Low concentrations of plant and fungal toxins induce recombinant gene expression

**Figure 1:**
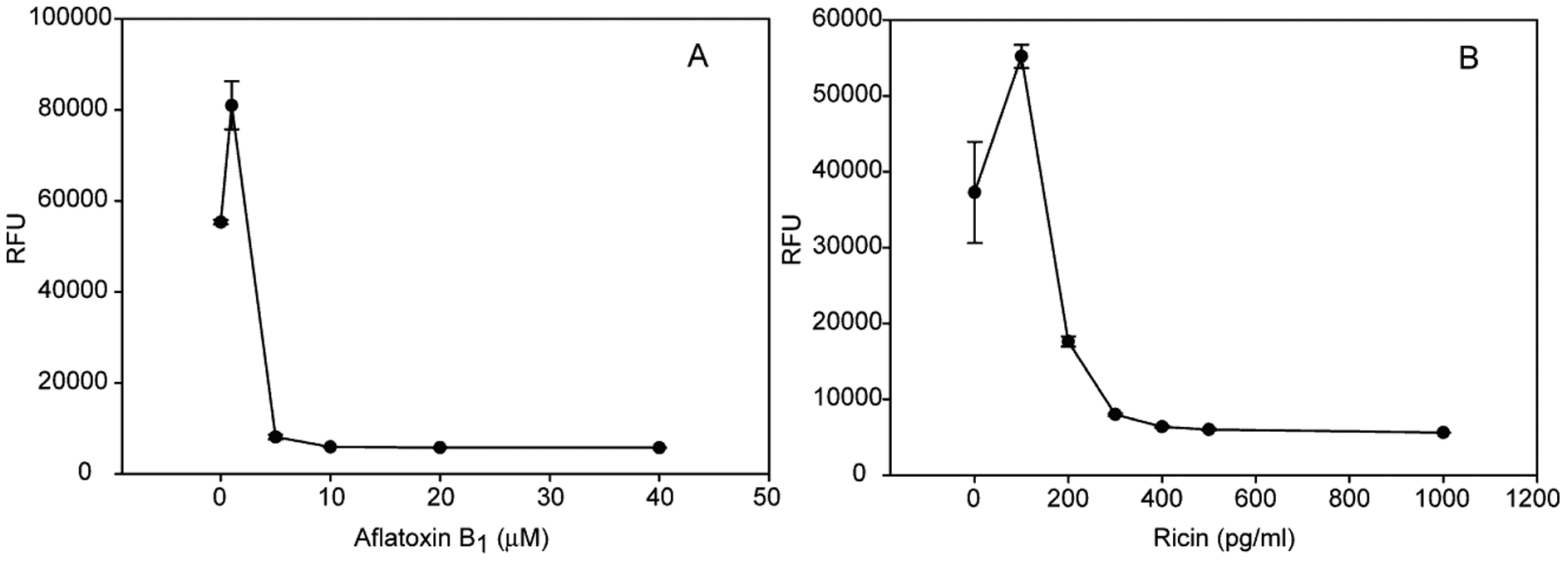
Increased GFP expression in Vero cells in the presence of low concentrations of AFB1 and ricin. Vero cells transduced with Ad-CMV-GFP were treated with increasing concentration of AFB1 (A) or ricin (B) toxins. GFP expression was quantified fluorometrically. Error bars represent standard errors. (n = 3).

We determined that changing the optimal cell culture medium by adding increasing concentrations of the plant toxin ricin or the fungal toxin AFB1 would enhance the expression of recombinant green fluorescent protein (GFP) production in Vero cells transduced with adenovirus-green fluorescent protein (Ad-GFP) following 48 h incubation. Our results (Figure 1) show that with the virus added to the cells simultaneously with AFB1 or ricin, the low toxin concentrations enhanced transcription and recombinant protein production. The enhancement does not follow a linear dose-response relationship between AFB1 or ricin concentrations and recombinant protein production. Specifically, 1 µM of AFB1 added to the Vero cells increased the fluorescence intensity associated with GFP by 46% from 55332±470 relative fluorescence units (RFU) to 80988±5279 RFU. The corresponding increase caused by the addition of 100 pg/ml ricin was 48%, from 37279±6645 RFU to 55214±1527 RFU. By contrast, higher levels of ricin or AFB1 toxin inhibited protein production and damaged the cells beyond their limits of recovery. Therefore, we cannot extrapolate the yield of recombinant protein production induced by low toxin concentration to predict protein production using higher concentrations.

### Extended AFB1 pre-incubation increases gene expression

**Figure 2:**
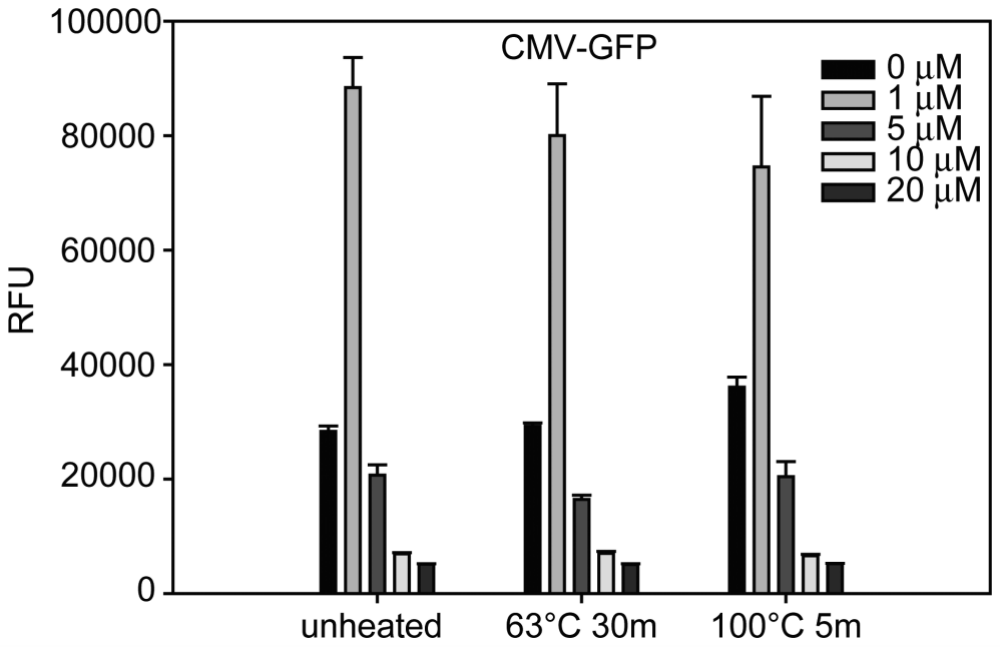
Induced GFP expression in Vero cells pre-incubated for 48 h with unheated or heated AFB1. A series of increasing concentrations of unheated or heated of AFB1 were incubated for 48 h with Vero cells. The cells were then transduced with Ad-CMV-GFP for 48 h. GFP expression was quantified fluorometrically. Error bars represent standard errors. (n = 3).

We performed two experiments to determine whether (a) recombinant protein yield is increased by a longer pre-incubation time of the cells with AFB1; and (b) heat treatment of AFB1 at 63°C for 30 min or 100°C for 5 min would result in AFB1 interacting differently with the cells than native AFB1 that may result in changes to recombinant protein transcription. A series of increasing concentrations of unheated or heated AFB1 were incubated for 48 h with Vero cells. The cells were then transduced with Ad-GFP for 48 h. Increasing the incubation time with the toxin to 48 h at the low concentration of 1 µM resulted in a 212% increase in fluorescence intensity from 28336±945 RFU to 88395±5238 RFU, indicative of higher recombinant protein production by the transduced cells (Figure 2). The results also show that (a) cells incubated for 48 h with AFB1 at the low concentration of 1 µM induced a significant increase (P<0.05) in recombinant protein production; (b) higher toxin concentrations inhibited protein production; and (c) heating the AFB1 did not affect protein production.

### Milk induces recombinant gene expression

We previously showed that milk can decrease cell viability in a dose-dependent manner [7]. We therefore wished to determine whether changing the optimal cell culture medium by adding milk would result in enhanced expression of recombinant GFP in Vero cells transduced with Ad-GFP.

**Figure 3:**
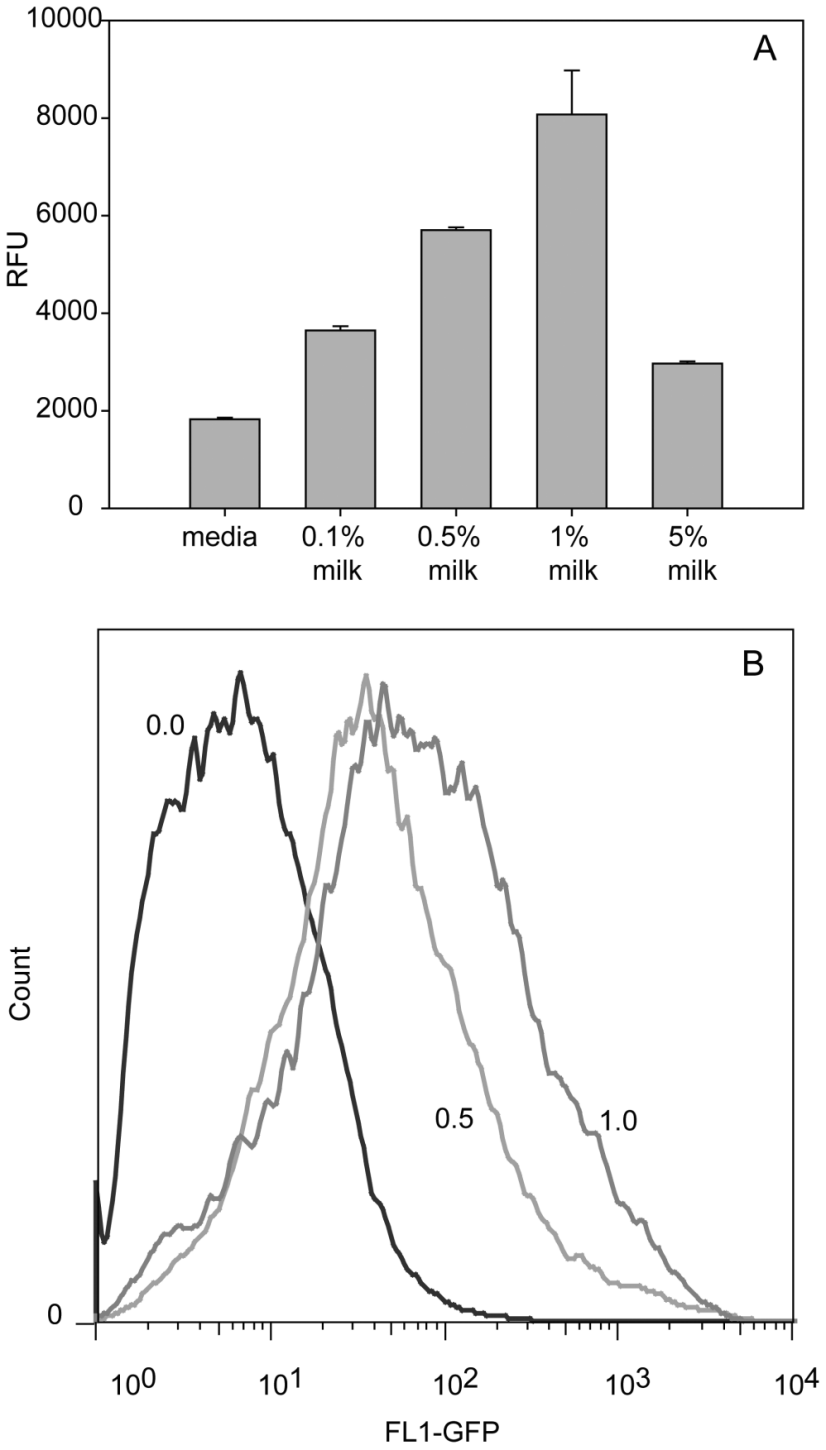
Effect of milk on recombinant gene expression in Vero cells. A series of increasing concentrations of reconstituted milk were incubated with transduced Vero cells with Ad-CMV-GFP. GFP expression was quantified fluorometrically (A) or by analysis of individual cells by flow cytometry. (B). Milk at a concentration of 1% dramatically increased the recombinant protein transcription. Note the shift in Figure 3B in the number of cells exhibiting higher cellular fluorescence. (n = 3).

As shown in Figure 3A, added milk at concentrations of 0.1%, 0.5%, 1%, and 5% significantly enhanced (P<0.05) gene expression as indicated by increased fluorescence intensity from 1828±30 for the control to 3648±84, 5705±52, 8072±903, and 2967±45 RFU or by 36% (control), 100%, 212%, 342%, and 62%, respectively. The highest increase was obtained with 1% milk. The somewhat lower increase in the presence of 5% milk is probably due to the observed adverse effects on the cells.

We used single cell analysis to determine whether increases in the fluorescence intensity were due to larger number of cells expressing GFP or whether individual cells exhibited higher levels of GFP expression. Flow cytometry analysis of 30,000 cells per sample shows that (a) milk at concentrations of 0.5% and 1% enhanced recombinant protein production in a dose-dependent manner; and (b) after the milk treatment, more cells expressed higher levels of GFP, as indicated by increased relative fluorescence values, with the fluorescence histogram shifting to the right (Figure 3B).

### The combination of ricin and milk increases recombinant GFP protein production

In a previous related study, we demonstrated that a component of milk has a high binding affinity to ricin [8]. Those results suggested that the reduction of the biological activity of ricin was due to the reduction in the amount of toxin available to galactose cell-surface receptors, decreasing the amount of toxin uptake by the cells.

In the present study we found that adding low concentrations of milk or ricin alone enhanced expression of recombinant proteins. We were therefore interested to find out whether the combination of 5% milk with low concentration of unbound ricin would increase recombinant protein expression. Increasing concentrations of ricin, which did not reach the saturation point and maximum binding capacity in milk, were added to milk and incubated with Vero cells transduced with Ad-GFP.

**Figure 4:**
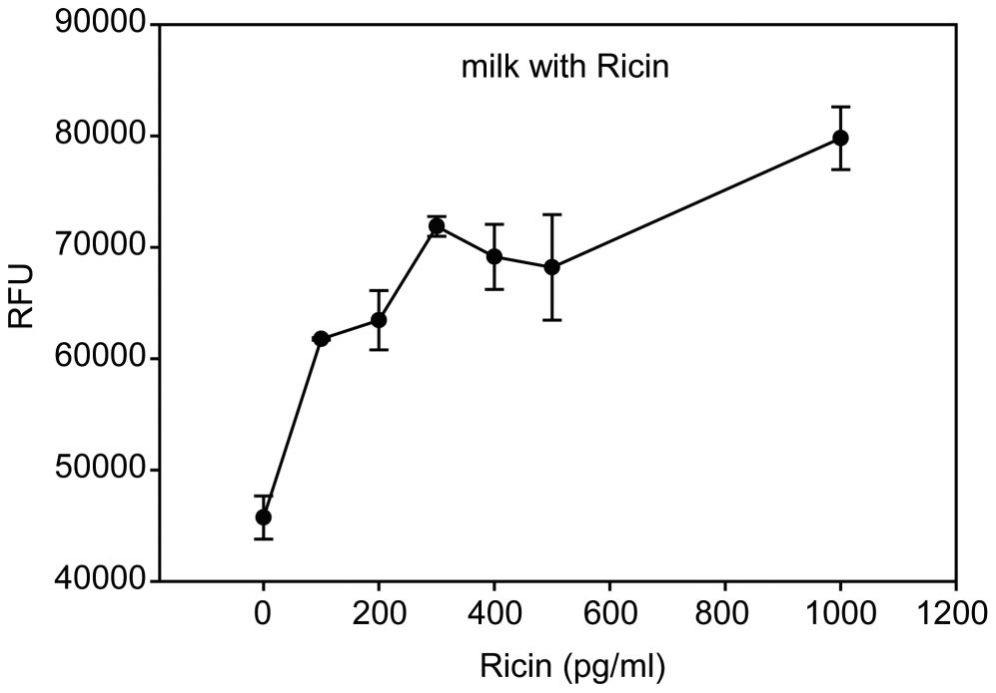
GFP expression in Vero cells induced by ricin with and without milk. GFP transduced Vero cells were treated with increasing concentrations of ricin in milk. After incubation of Vero cells with Ad-GFP and ricin for 3 days, GFP expression was quantified fluorometrically. Error bars represent standard errors. (n = 3).

Our results (Figure 4) show that adding ricin at concentrations of 100, 300, and 1000 pg/ml to milk results in a significant increase in levels (P<0.05) of GFP expression. The fluorescence intensity increased from 45738±1942 to 61769±112, 71884±885, and 79791±2812 RFU in a concentration-dependent manner, corresponding to increases of 35%, 57%, and 74%, respectively. The results also show that milk reduces the amount of toxin available to the cells and that the same high concentrations of ricin without added milk killed the cells (Figure 1).

### Enhanced expression of recombinant β-galactosidase and firefly luciferase

Additional studies were carried out designed to determine whether (a) changing the optimal cell culture medium would enhance expression and production of recombinant proteins other than GFP; (b) there is a correlation between mRNA and protein levels determined as signal intensities produced after substrate is added to the active enzyme; and (c) other coding regions that regulate gene expression in addition to the cytomegalovirus promoter (CMV), would respond to this change and would initiate translation and expression of recombinant proteins. To quantitatively monitor gene expression, we constructed the following additional three adenovirus vectors that encode and express measurable recombinant proteins: Lac-Z gene under the control of the CMV or Rous Sarcoma Virus (RSV) promoter and luciferase gene under the control of the CMV promoter.

**Figure 5:**
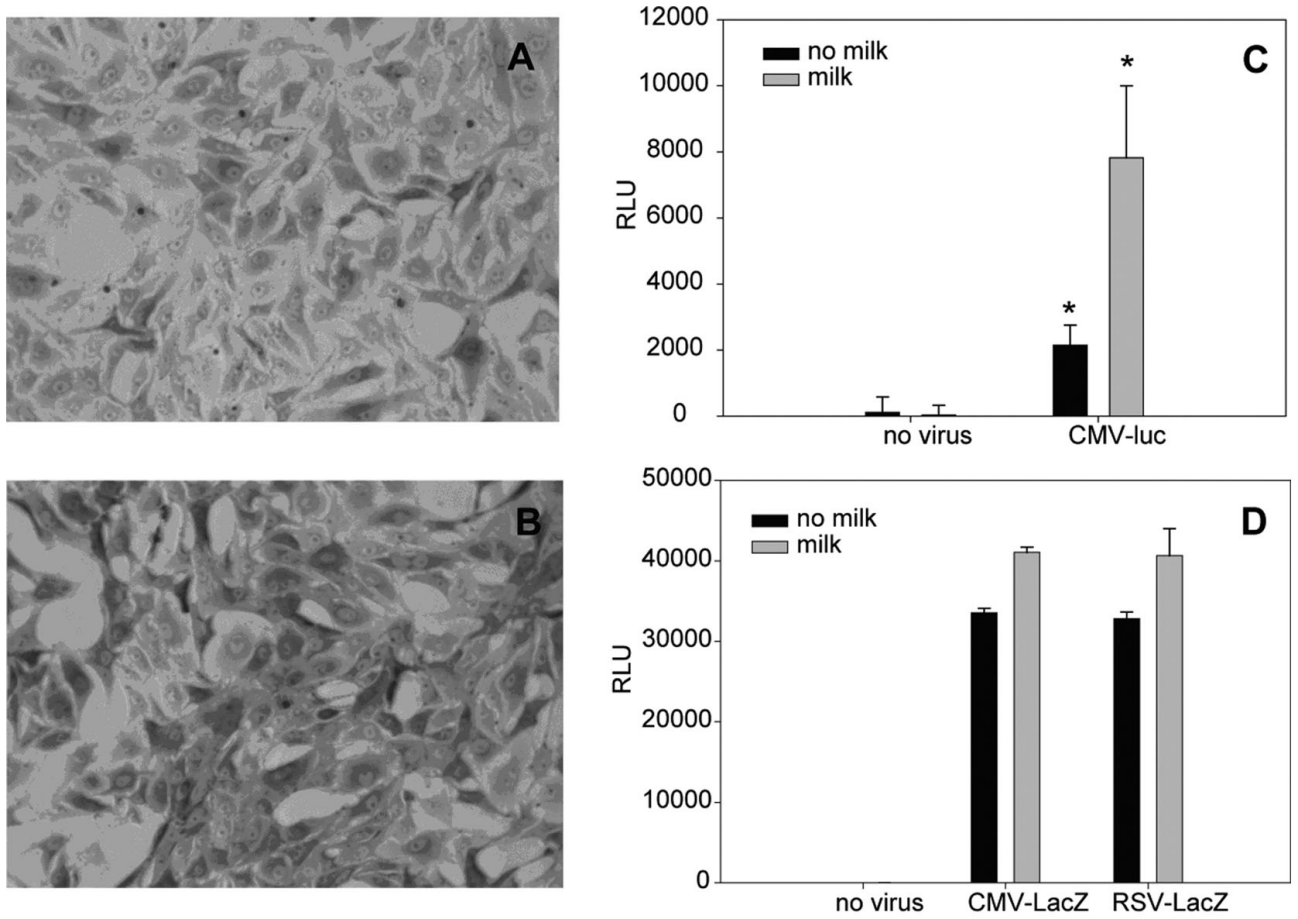
Milk enhances the expression of β-galactosidase and luciferase enzymes. Control transduced Vero cells (without milk) (5A) and cells with 1% milk (5B) were incubated for 24 h. The cells were then fixed with glutaraldehyde followed by addition of x-gal solution. After 10 min incubation, the cells were analyzed by bright field light microscopy ×200 magnification. Vero cells transduced with Ad-CMV-Luc (5C) with and without 1% milk were incubated for 24 h. After addition of substrate, the relative light units (RLU) emitted were measured with a luminometer. Vero cells with and without 5% milk were transduced with Ad-CMV-LacZ or Ad-RSV-LacZ (5D) and RLU were then measured after addition of substrate. Error bars represent standard errors. (n = 3).

As shown in Figure 5 (A–B), bright field light microscopic analysis visually shows that adding 1% milk also enhanced the expression of the recombinant protein β-galactosidase. The blue color intensity level of the cells (darker grey in the photographs shown) indicates that β-galactosidase protein expression was higher in treated cells (B) than in the control (A).

The 1% milk solution also significantly enhanced (P<0.05) the expression of the recombinant luciferase activity (Figure 5C) measured by relative light units (RLU) emitted after the luciferin substrate was added. The observed increase in RLU was from 2151±605 to 7818±2182, corresponding to an increase of 263%.

### Effects of the promoter on protein expression level

As shown in Figure 5D, there was no significant difference between CMV and RSV promoter activity in their abilities to enhance the expression level of the recombinant β-galactosidase after treatment with 5% milk. Milk at concentration of 5% enhanced the expression level from 33547±543 to 41051±651 RLU or by 22% with the CMV promoter and from 32804±809 to 40648±3337 RLU or by 24% with the RSV promoter.

### Low levels of environmental stress increase mRNA levels of the recombinant genes

**Figure 6:**
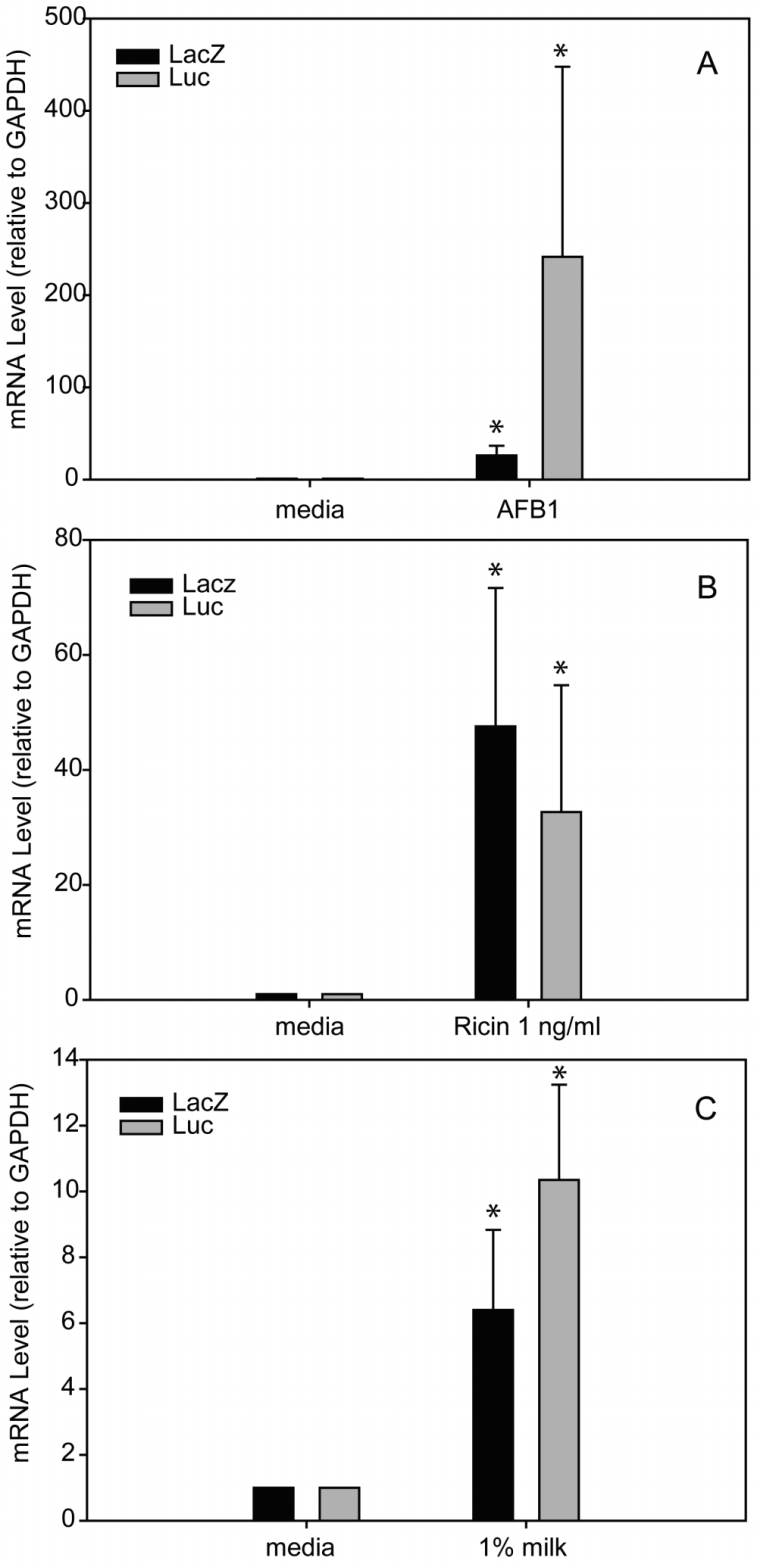
mRNA expression of Lac-Z gene and LUC gene measured by qRT-PCR. AFB1 was incubated for 1 day with Vero cells then transduced with Ad- LacZ or Ad- LUC for 2 days (A). Vero cells were transduced with Ad- LacZ or Ad- LUC treated with 1 ng/ml ricin (B) or 1% milk (C). Error bars represent standard errors, and values significantly different from control are marked with an asterisk (P<0.05). (n = 3).

The data at Figure 6 show that treatment with AFB1, ricin, and 1% milk enhanced transcription level of the recombinant genes and that there is a correlation between luciferase and β-galactosidase mRNA levels and the reporter gene activity. Stimulation with milk resulted in a 6.4-fold increase in β-galactosidase mRNA levels. The corresponding increase in luciferase mRNA transcription was 10.3-fold. Stimulation with ricin resulted in 47.5-fold increase in β-galactosidase mRNA levels. The corresponding increase in luciferase mRNA transcription was 32.7-fold. Stimulation with AFB1 resulted in 241-fold increase in β-galactosidase mRNA levels. The corresponding increase in luciferase mRNA transcription was 26-fold.

### Quantitation of Viral DNA in Transduced Vero Cells

**Figure 7:**
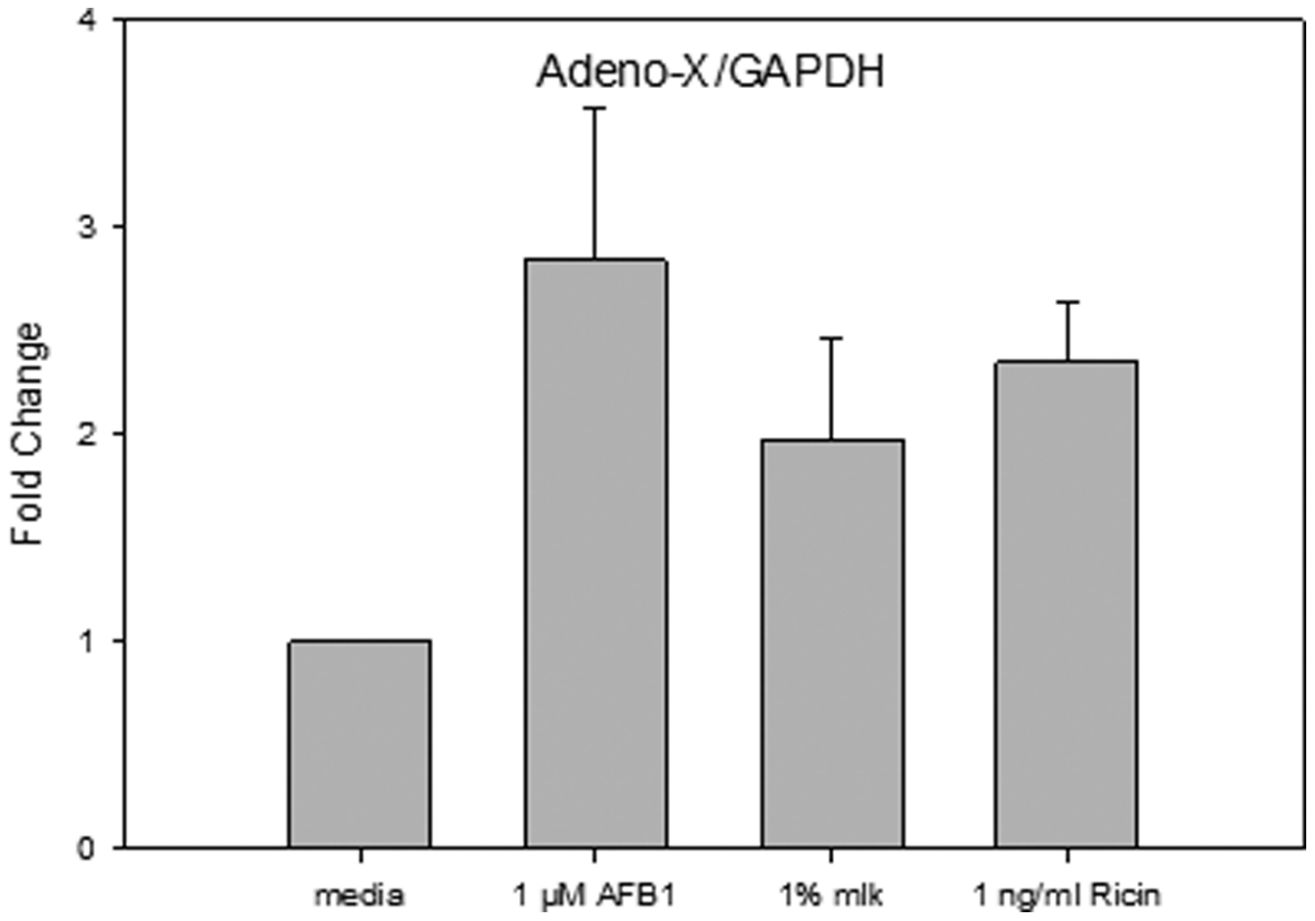
Quantitation of viral DNA in transduced Vero cells. Vero cells transduced with Ad-CMV-GFP were treated with 1 µM AFB1, 1% milk, or 1 ng/ml ricin. The PCR product for the 167 base pairs amplicon was quantified by qPCR. Error bars represent standard errors. (n = 3).

Adenovirus enters cells via a receptor-mediated endocytosis following binding to the cell surface protein CAR [9]. We wished to determine whether the observed enhancement of recombinant protein expression in transduced Vero cells by low levels of AFB1, milk, or ricin is due to an increase in efficiency of viral infectivity. Our results (Figure 7) show that by using the Clontech Adeno-X PCR primers we were able to quantitate virus DNA in the transduced Vero cells. The results also show that treatment of the cells with the three stressors (1 µM AFB1, 1% milk, or 1 ng/ml ricin) caused an increase in the Adeno-X DNA levels by 2.8390±0.7430, 1.9760±0.4820 or 2.3510±0.2840 fold, respectively. The increased levels are, however, not statistically significantly enhanced (P>0.05). We, therefore, cannot conclude that the observed increase in viral infectivity is fully or partly responsible for the observed increase in the recombinant proteins production.

## Discussion

To our knowledge this is the first report demonstrating that supplementing tissue culture medium with small doses of ricin, AFB1, or milk enhances recombinant protein production expressed in mammalian cells. The 1% milk solution is more effective than AFB1 or ricin. It enhanced recombinant protein synthesis by more than three-fold (Figure 3). The enhancement, however, was not uniform. It varied widely with the nature and concentration of the stressor and the protein being expressed. Our data show that cell passage number or cell division number affects protein expression levels. This observation is consistent with the data of Siissalo et al [10], therefore, in the present study each set of experiments was performed with cells from the same passage on the same day. All figures show significant differences between control and treated samples. We do not know the exact molecular mechanism of how low levels of stress alter cell physiology and enhance recombinant protein production. We speculate that the cells react these low levels of stress stimuli to adapt to the environmental changes by enhancing transcription of genes that alter cell physiology to increase levels of protective molecules and enhance the capability of cellular systems involved in adaptation and cell survival. Our results show that low levels of stress significantly increase transcription efficiency and mRNA levels (Figure 6). The consequence of these events is an increased rate of protein synthesis (Figures 1–5). However, increase in mRNA levels was more than protein abundance. This observation is consistent with the data of Guo et al. [11] who found that mRNA expression patterns could be accompanied by a 20-fold lower protein expression.

Because there are many complicated and varied post-translational mechanisms involved in translating mRNA into protein that are not yet sufficiently known, we were not able to calculate protein concentration from mRNA levels.

Moreover, the proposed biochemical changes do not, however, follow a linear dose-response relationship. Higher levels of stress-inducing conditions have an opposite effect. They inhibit recombinant protein production, presumably because higher stress levels damage the cells beyond their limits of recovery and the cell cannot overcome the damaging effect resulting in observed cell death (Figure 1). To demonstrate the generality of the approach we analyzed the effect of high concentration of the AFB1 stressor on Vero cell viability, as well as toxicity, using MTT assay and MultiTox-Glo Multiplex Cytotoxicity Assay-Promega. The results (not shown) were similar to those obtained with the GFP fluorescence assay.

A anonymous reviewer pointed out that the 1.46-fold increase in protein production in the Vero cells induced by 1 µM AFB1 and the corresponding 1.48-fold induced increase by ricin can be explained the observed increase in the infectivity of the adenovirus. By contrast, the increase in protein expression that cannot be explained by an increase on adenovirus infectivity is the observed 4.4-fold expression induced by addition of 1% milk. The situation is even more complicated because milk at the 5% concentration induced a significantly lower expression than at the 1% level (Figure 3). We have no obvious explanation for these effects of milk, except to suggest that the higher concentration of milk might adversely affect the viability of the cells.

Other relevant studies describe dose dependent toxicity in humans or in animals receiving higher doses of the adenovirus vector that is used to enhance gene expression of the recombinant bioactive proteins in gene therapy [3]. The results presented here suggest that it may be possible to achieve higher levels of gene expression with lower virus titer to induce improved therapeutic results in target tissues. This is because combined lower virus titers and stress-inducing conditions will minimize the cytotoxic effect associated with high virus titers.

In this study we overexpressed three different recombinant proteins. The positive findings suggest that the method has the potential to facilitate *in vitro* production of bioactive proteins used in agriculture and therapy. Although introduction of the stressor can contaminate the therapeutic recombinant protein, immunoseparation may be applied to eliminate this problem.

Our results also show that even at low concentration, AFB1 enhanced transcription and protein production. These results may have implications for the consumption of food treated with various reagents intended to reduce AFB1 content. For example, even though the treatment of maize grain with citric acid reduced AFB1 and AFB2 by 96.7% [12], it is likely that the residual low aflatoxin levels may have unknown biological effects. The consequences of consuming food with low AFB1 content merit study [13], [14], [15], [16], [17].
